# Research on Bond Participants’ Emotion Reactions Toward the Internet News in China’s Bond Market

**DOI:** 10.3389/fpsyg.2022.855063

**Published:** 2022-03-29

**Authors:** Wei Zhang, Jun Wang, Mu Tong

**Affiliations:** ^1^School of Economic Information Engineering, Southwestern University of Finance and Economics, Chengdu, China; ^2^Collaborative Innovation Center of Financial Security, Southwestern University of Finance and Economics, Chengdu, China

**Keywords:** emotion reaction, financial news, bond market, bond investor, credit rating agency

## Abstract

The literature has widely studied the market response to the financial news or events but mainly focused on the stock market. This article associates the concept of internet news with the bond market response and attempts to examine how credit rating agencies (CRAs) and bond investors, two important bond participants, react to financial news on the internet with a range of multiply regressions. Our empirical study leads to several findings. First, CRAs tend to ignore the warnings of financial news on the internet, whereas bond investors strongly react to such news. Second, there is an asymmetry in bond investors’ reactions to good news compared to bad news, with investors being more sensitive to bad news. Third, there is heterogeneity in the psychological reaction where bond investors do not react to the news about central state-owned enterprises (SOEs) but to the news about other enterprises. Finally, there is an asymmetric response driven by news timeliness that bond investors are more sensitive to the latest news articles than old ones. Overall, our study confirms the existence of psychological reactions to the financial news on the internet in China’s bond market, which has significance for keeping bond market participants from overreacting or underreacting to market news.

## Introduction

China has been the second-largest bond market globally, with an annual issuance of 57.3 trillion RMB in 2020, significantly benefiting the Chinese economy to achieve rapid growth. Despite the fast development, the information disclosure in China’s bond market is still relatively immature. For example, managers tend to disclose some good information but delay the announcement about bad information (Tang et al., 2018). There is limited information available about the financial market, making the market more susceptible to rumors (Chen and Haga, 2021).

In the digital economy era, the importance of financial news on the internet, a new carrier of information transmission, is increasing. It can be seen as a supplement to information disclosure because some information contained in the financial news can give early warnings before official announcements, predict the firm performance in the future, and provide clear expectations about the inherent risks (Grewal et al., 2019). On the other hand, once an investment decision is emotionally made based on the financial news on the internet, investors may suffer wealth loss or missing investment opportunities. Studies from the behavioral finance theory reveal that the price of any assets depends on the market participants’ emotion reactions to information or news (Thaler, 2019). It needs to be emphasized that the idea that emotional reactions might influence asset prices is not a prerogative of behavioral finance theory. Psychological studies also argue that emotional responses are ubiquitous and may significantly depart from cognitive responses when facing uncertain information and news (Kahneman and Tversky, 2013). In general, biased emotions, general knowledge, and experience might account for investor reactions to information or news (Fischer, 2011), and similar studies have been mainly tested in the stock market. For example, Cervellati et al. (2014) found that the stock price and trade volume reacted to the second-hand information in Italy. Suleman (2012) analyzed the stock market’s response to the political news, and their findings showed that good political news increased the stock return and decreased the volatility, while bad political news negatively impacted the abnormal returns and increased the volatility. However, little literature has focused on the bond market’s reaction to financial news. Therefore, how bond investors and CRAs, two vital participants in the bond market, react to financial news on the internet should be studied.

Abundant literature has revealed that it is unique for central state-owned enterprises (SOEs). First, the chairpersons of central SOEs in China were ministers or directly reported to ministers in planned economy days. Nowadays, they still have a considerable influence at different government levels in China, especially at the highest government level. At the same time, the Communist Party of China (CPC) and central government also exert tight control over central SOEs. Most senior managers of central SOEs are members of the CPC, and they are subjected to the discipline of the CPC (Andrews-Speed, 2010). Second, central SOEs get much stronger support for business from the government, and the government is more likely to take action to avoid default when central SOEs have high risks (Huang et al., 2020a). This implies that bond investors may react differently to the news about central SOEs and other enterprises, which has barely been investigated in existing studies. This article, therefore, will extend existing literature and investigate whether the news reaction is associated with enterprise ownership.

Some scholars have discussed that there is a decline in the news effect over time. For example, Zhao and Zeng (2019) pointed out that the forecasting ability of news related to short-scale trends and long-scale trends only lasted 1 week, whereas the distinguish trends could last up to 3 weeks. Chen et al. (2021) found that the sensitivity of informed investors to the policy news would diminish over time. Thus, this article extends previous literature and attempts to investigate whether bond participants have different reactions to new news and past news.

The goal in this article is attempt to answer three research questions. The first question is whether there is a bias in the emotion reactions to bad news and good news in China’s bond market and whether the emotion reactions are consistent with previous literature. The second question is whether bond investors’ reactions to financial news depend on enterprise ownership, or whether bond investors have a stronger emotional reaction to financial news about central SOEs than other enterprises. The third question is whether bond investors’ reactions to financial news depend on news timeless, or whether bond investors have stronger reactions to new financial news than old financial news.

We make several contributions to the literature in this study. First, investor reactions have been extensively examined (Cano et al., 2016; Narayan and Bannigidadmath, 2017; Chen et al., 2021) in the stock market, but we focused on the bond market, for which limited evidence is available. Second, prior literature mainly focused on the event news and macroeconomic level news, such as real estate event news (Chen et al., 2021), consumer price index (CPI) news (Schwert, 1981), unemployment rate (UR) news (Hakkio and Pearce, 1985), and producer price index (PPI) news (Beber and Brandt, 2010). Instead, we are interested in the financial news on the internet at each specific-enterprise level, which has a higher dimension and contains more information. Finally, we examine the heterogeneous response driven by enterprise ownership and news timeliness that bond investors differently react to central SOE news, other enterprise news, old news, new news.

The layout of the paper is organized as follows. Part 1 is the introduction. Part 2 reviews the literature. Part 3 illustrates the research design. Part 4 presents and discuss the empirical results. Part 5 offers the robust check, whereas we conclude in Part 6.

## Literature

### Financial News and Credit Rating Agencies

Credit ratings, viewed as CRAs’ reactions toward credit risk valuation, play an important role in the bond market because they correct the information asymmetry between bond issuers and financial markets (Bongaerts et al., 2012). Therefore, bond participants highly rely on credit ratings and view them as the license to the bond market (Partnoy, 2006). Abundant literature has studied the determining factors in credit ratings. For example, Altman et al. (1977), Xia (2014), and de Vries and de Haan (2016) pointed out that firms with better financial performance were more likely to receive high credit ratings. Bhandari and Golden (2021) found that CRAs had a stronger response to CEO’s political ideology. Camanho et al. (2020) revealed that CRAs tended to upgrade credit ratings when facing fierce competition in the credit rating market. However, there is little literature available for how CRAs or credit ratings react to financial news on the internet. Two representative studies conducted by Tsai et al. (2010, 2016) found that the text information from the newspapers contained incremental informational content for credit risk evaluation. Therefore, they concluded that CRAs had a significant reaction to the information from the newspapers. Lu et al. (2012) revealed that news was helpful to predict future credit ratings, implying that CRAs significantly reacted to the coverages in the newspaper. In this article, we will extend the previous literature to investigate the attitudes of CRAs to the financial news on the internet.

### Financial News and Bond Investors

Abundant studies have pointed out that financial news plays an important role in the financial market, and those financial news coverages are widely used and analyzed by investors (Sabherwal et al., 2011; Narayan and Bannigidadmath, 2017; Du, 2020). Narayan and Bannigidadmath (2017) examined how investors reacted to the financial news on Islamic and conventional stock markets from 2005 to 2012. They found that both positive and negative news influenced stock returns, but positive news had a relatively larger impact on stock investors. However, Du (2020) got the opposite conclusion that stock investors in Japan had a stronger reaction to negative coverages. Based on 130 oil-related words, Loughran et al. (2019) investigated how investors responded to the media news on the oil market. Their findings showed that investors often overreacted to oil news. Corbet et al. (2020) examined the relationship between news coverages and Bitcoin investors’ reactions, and they found that bitcoin investors were not sensitive to CPI and GDP news but had a significant reaction to unemployment and durable goods news.

Recently, a handful of literature has focused on how the bond market reacted to financial news (Beber and Brandt, 2010; Defond and Zhang, 2014; Caporale et al., 2018). According to the contract theory, investors in the bond market were more conservative than investors in other financial markets (Kothari et al., 2010), implying that investor emotions in the bond market may be more sensitive to news coverages. Defond and Zhang (2014) tested how bond investors reacted to good and bad earning news. Their findings showed that bond investors were more likely to impound bad earning news on a timelier basis than stock investors. Beber and Brandt (2010) studied how investors responded to positive and negative macroeconomic news during economic expansions and recessions. They found that bond investors strongly responded to negative news associated with non-farm payrolls in expansions and positive news associated with inflation in recessions. Using a VAR-GARCH model, Caporale et al. (2018) investigated how bond investors reacted to macro news in the euro area from 1999 to 2014. Their findings showed that negative coverage associated with macro news positively affected bond investors in Greece, Ireland, Italy, Portugal, and Spain, and the responses became stronger in the 2008 financial crisis. However, there are several limitations in those studies. First, news used in existing literature mainly focused on the macroeconomic level, but seldom literature focused on the financial news on the internet. Second, previous literature mainly focused on the investor reactions in the secondary market rather than the investor reactions in the primary market. Therefore, this proposed article focuses on bond investors’ reactions toward the financial news on the internet at each enterprise-specific level in the primary bond market.

Previous literature has discussed the difference between central SOEs and other enterprises. For example, Zhao (2006) pointed out that central SOEs play an important role and enjoy many privileges in China’s economy. Lin et al. (2020) argued that central SOEs were viewed as a way to maintain social stability, without which the economy could not function properly. Ralston et al. (2006) analyzed different enterprise ownership types and concluded that SOEs contributed a significant share to the total output, particularly true for the sectors with strategic value. However, no literature associated the concept of enterprise ownership with the financial news on the internet. Considering that investor psychologies and stock market behaviors have different reactions to different news (Abreu and Mendes, 2012), we will extend previous literature and examine whether bond investors have a stronger emotion reaction to news about other enterprises than SOEs.

News timeliness has been studied extensively in previous literature. For example, Defond and Zhang (2014) examined the timeliness of the market reaction to earnings news, and they found that the timeliness of bond investors’ reactions to bad news was concentrated primarily among speculative-grade bonds. Matsubara et al. (2012) and Chen et al. (2021) concluded that the news influence would diminish over time. Eachempati and Srivastava (2021) argued that investors were inclined to ignore old news that may not stir the market sentiments, but they were sensitive to new news. In this article, our study will further analyze whether bond investors have different reactions to new news and old news.

## Methodology

### Measure of the Reactions From Market Participants

If bond investors and CRAs have emotion reactions toward the bad (good) news, we will observe lower (higher) credit ratings or higher (lower) bond yield spreads. Thus, we employ credit ratings and bond yield spreads as the proxy variables of CRA and bond investor reactions.

The credit rating can be divided into two types. One is the bond project rating (*BR*), which can be regarded as the security of the bond itself. The other is corporate entity rating (*CR*), which can be viewed as the overall security of the bond issuer. Following Xia (2014) and Huang et al. (2020b), we employ the numerical value to quantify *BR* and *CR* as: AAA = 1, AA+ = 2, AA = 3, AA− = 4, A+ = 5, A = 6, A− = 7, BBB+ = 8, BBB = 9, BBB− = 10, BB+ = 11, BB = 12, BB− = 11, B+ = 12, B = 13, B− = 14, and lower than B− = 15.

Following Qian (2018) and Chen et al. (2021), the bond yield spread can be measured by the difference between the corporate bond yield and government bond yield, which is given as follow:


(1) $$Y\,i\,e\,l\,d\,S\,p\,r\,e\,a\,d=y\,i\,e\,l\,d_{1}-y\,i\,e\,l\,d_{2}$$

where _*yield_1*_ is the corporate bond yield, _*yield_2*_ is the government bond yield with the same maturity as the corporate bond.

### News Quantification

#### News Classification

We will classify the news into three types: positive, negative, and neutral news. First of all, three financial researchers with professional knowledge are invited to mark 100,000 news articles manually. Following Das and Chen (2007), we then apply three classifiers, namely polynomial naïve Bayes, support vector machine (SVM), and random forest (RF), to train the marked news that is categorized manually by three experienced researchers. Finally, we employ the trained classifiers to label the unmarked news articles, and the voting scheme is used to classify the unmarked news^1^.

#### Measure of News Sentiment

After classifying the news type, two indexes are introduced to measure the news sentiment from *T*_1_ (6 months prior to the bond issue date) to *T*_0_ (the bond issue date), which is denoted by:


(2-1) $$B\,a\,d\,R\,a\,t\,i\,o=\frac{N}{T}$$

(2-2) $$G\,o\,o\,d\,R\,a\,t\,i\,o=\frac{P}{T}$$

where *T* is the total number of news articles over the 6-month window, *N* and *P* are the total number of bad and good news over the 6-month window, respectively.

Next, we introduce the concept of positive/negative day to avoid false conclusions because repeated news articles could be released on different news websites on the same day. Specifically, we view the day as a positive or negative day for one specific enterprise when the enterprise is exposed to more good or bad news on one day.

Finally, to improve the precision, another two sentiment proxies are introduced to measure the proportion of bad and good news articles at the day level, which is given as follow:


(3-1) $$D\,a\,y\,B\,a\,d\,R\,a\,t\,i\,o=\left\{ \begin{array}{c} \frac{T\,N\,D}{D}\,\,i\,f\,\,D>0\\ 0\,\,i\,f\,\,D=0\, \end{array}\right.$$

(3-2) $$D\,a\,y\,G\,o\,o\,d\,R\,a\,t\,i\,o=\left\{ \begin{array}{c} \frac{T\,P\,D}{D}\,\,i\,f\,\,D>0\\ 0\,\,i\,f\,\,D=0\, \end{array}\right.$$

where *D* is the total number of days in which the news is released on the internet for a specific enterprise, *TND* is the number of negative days for a specific enterprise, *TPD* is the number of positive days for a specific enterprise.

### Econometric Models

To examine whether CRAs and bond investors have emotion reactions to financial news on the internet, we use following multiple regressions:


$$B\,R=\alpha +\beta_{0}\,X+\beta_{1}\,L\,n\,T\,o\,a\,l\,N\,e\,w\,s+F\,i\,r\,m\,C\,o\,n\,t\,r\,o\,l+$$

(4) B⁢o⁢n⁢d⁢C⁢o⁢n⁢t⁢r⁢o⁢l+I⁢n⁢d⁢u⁢s⁢t⁢r⁢y+Y⁢e⁢a⁢r+ε

$$Y\,i\,e\,l\,d\,S\,p\,r\,e\,a\,d=\alpha +\beta_{0}\,X+\beta_{1}\,L\,n\,T\,o\,a\,l\,N\,e\,w\,s+\beta_{2}\,C\,R+F\,i\,r\,m$$

(5) C⁢o⁢n⁢t⁢r⁢o⁢l+B⁢o⁢n⁢d⁢C⁢o⁢n⁢t⁢r⁢o⁢l+R⁢a⁢t⁢e⁢C⁢o⁢n⁢t⁢r⁢o⁢l+I⁢n⁢d⁢u⁢s⁢t⁢r⁢y+Y⁢e⁢a⁢r+ε

where *X* is the variable related to news sentiment indexes, including *BadRatio*, *GoodRatio*, *DayBadRatio*, and *DayGoodRatio.*_*LnToalNews*_ is the nature logarithm of the total number of news articles. *CR* is the corporate entity rating. *Firm Control* includes the natural logarithm of total assets, asset-liability ratio, total assets turnover, current ratio, return on equity, and enterprise ownership. *Bond Control* includes bond size, bond maturity, collateral clause, and bond type. *RateControl* includes two yields: 1-year government bond yield and government bond yield with the same maturity as the corporate bond. Year and industry fixed effects control specific time and industry factors. Table 1 describes these variables in detail.

**TABLE 1:** Variable definition.

Variable	Definitions
**Dependent variables**	
CR	Corporate entity rating (AAA = 1, AA+ = 2, AA = 3, AA− = 4, A+ = 5, A = 6, A− = 7, BBB+ = 8, BBB = 9, BBB− = 10, BB+ = 11, BB = 12, BB− = 11, B+ = 12, B = 13, B− = 14, and lower than B− = 15)
BR	Bond project rating (AAA = 1, AA+ = 2, AA = 3, AA− = 4, A+ = 5, A = 6, A− = 7, BBB+ = 8, BBB = 9, BBB− = 10, BB+ = 11, BB = 12, BB− = 11, B+ = 12, B = 13, B− = 14, and lower than B− = 15)
YieldSpread	The difference between the government bond yield and the municipal corporate bond yield, with the same maturity
Gua	Whether the bond issuer seeks the credit guarantee or not?
**Firm Control**	
LnAsset	The natural logarithm of MC’s total assets with one lagged year
Liability	The asset-liability ratio with one lagged year
Current	The current ratio with one lagged year
ROE	The return on equity with one lagged year
TurnOver	The total assets turnover with one lagged year
SOE	The enterprise ownership (central SOEs and other enterprises)
**Bond Control**	
BondSize	Bond size
Maturity	Bond maturity
Collateral	Can the bond be collateral (Yes: 1; No: 0)?
BondType	Bond Type^1^ (including the enterprise bond, corporate bond, and medium-term note)
**Rate Control**	
GovYield	Government bond yield with the same maturity as the corporate bond
OneYearRate	1-year government bond yield
**News variable**	
BadRatio	See Part News Quantification
GoodRatio	See Part News Quantification
DayBadRatio	See Part News Quantification
DayGoodRatio	See Part News Quantification
LnTotalNews	The natural logarithm of total news articles over 6 months
**Other control variable**	
Industry	Industry
Year	Year

*^1^Enterprise bonds approved by the National Development and Reform Commission (NDRC) are traded in the exchange and interbank markets. Corporate bonds approved by the China Securities Regulatory Commission (CSRC) are traded in the exchange market.*

### Data Statistics

We collect the data from several sources, ranging from 2010 to 2020. Data related to bond and firm characteristics are downloaded from Wind^2^. 275,470 news articles are collected from China’s mainstream financial news websites using web-crawler technology. Deleting some observations with missing information, we finally select 2,510 bonds (central SOEs issue 690 bonds and other enterprises issue 1,820 bonds) issued by listed enterprises in China, excluding financial bonds, convertible bonds, notes, and asset-backed securities. Tables 2, 3 report the statistical results based on Stata 15.

**TABLE 2:** Descriptive statistics of the internet news about China’s listed enterprises from 2010 to 2020.

	Panel A: All sample			Panel B: Central SOEs			Panel C: Other enterprises		
Variable	*N*	Mean	p50	*N*	Mean	p50	*N*	Mean	p50
BR	2394	1.772	1	682	1.268	1	1712	1.973	2
CR	2510	2.022	2	690	1.362	1	1820	2.271	2
YieldSpread	2510	224.0	193	690	138.8	132	1820	256.2	239
TotalNews	2510	91.37	19	690	133.9	45	1820	75.25	16
LnTotalNews	2510	3.146	2.944	690	3.599	3.806	1820	2.974	2.773
BadRatio	2510	0.181	0.146	690	0.192	0.167	1820	0.177	0.143
GoodRatio	2510	0.683	0.714	690	0.664	0.691	1820	0.691	0.721
DayBadRatio	2510	0.209	0.177	690	0.222	0.200	1820	0.204	0.167
DayGoodRatio	2510	0.679	0.700	690	0.666	0.667	1820	0.684	0.714
									

**TABLE 3:** Pearson correlation coefficients.

	YieldSpread	CR	LnTotalNews	BadRatio	GoodRatio	DayBadRatio	DayGoodRatio
YieldSpread	1						
CR	0.575	1					
LnTotalNews	–0.184	–0.300	1				
BadRatio	0.0103	0.0473	0.0340	1			
GoodRatio	–0.0009	–0.0051	–0.0168	–0.753	1		
DayBadRatio	0.0032	0.0273	0.0971	0.960	–0.736	1	
DayGoodRatio	–0.0162	–0.0204	0.0236	–0.747	0.949	–0.775	
LnAsset	–0.360	–0.749	0.373	–0.0613	0.0140	–0.0284	
Liability	0.0986	–0.0295	0.0603	–0.0632	0.0598	–0.0510	
ROE	0.0069	–0.115	–0.0024	–0.139	0.105	–0.141	
Current	0.191	0.268	–0.102	–0.041	0.0405	–0.0396	
TurnOver	–0.0147	–0.0241	0.0276	0.0404	–0.0353	0.0393	
Gua	0.150	0.358	–0.0574	0.132	–0.112	0.114	

	**DayGoodRatio**	**LnAsset**	**Liability**	**ROE**	**Current**	**TurnOver**	**Gua**

DayGoodRatio	1						
LnAsset	0.0258	1					
Liability	0.0515	0.216	1				
ROE	0.108	0.106	–0.266	1			
Current	0.0310	–0.286	–0.0302	0.0989	1		
TurnOver	–0.0252	0.0327	–0.0582	–0.0711	–0.251	1	
Gua	–0.102	–0.305	0.0228	–0.128	0.108	0.0404	1

Panel A of Table 2 reports the statistics about the whole news sample. The mean of *TotalNews* is 91.37, but the median is 19. *BadRatio* (mean of 0.181, median of 0.146) and *DayBadRatio* (mean of 0.209, median of 0.177) are relatively smaller than *GoodRatio* (mean of 0.683, median of 0.714) and *DayGoodRatio* (mean of 0.679, median of 0.700), indicating that more positive financial news is released on the internet.

Panel B of Table 2 provides the statistics about central SOEs. We find that central SOEs have relatively higher bond project ratings (mean of 1.268, median of 1), higher corporate entity ratings (mean of 1.362, median of 1), and lower bond yield spreads (mean of 224, median of 193). In addition, we also find that central SOEs have more positive news than negative news, which is consistent with Panel A.

Panel C of Table 2 presents the statistics about other enterprises. Consistent with Panel A and Panel B, other enterprises are also exposed to more good news. On the other hand, other enterprises have relatively lower bond project ratings (mean of 1.973, median of 2), lower corporate entity ratings (mean of 2.271, median of 2), and higher bond yield spreads (mean of 256.2, median of 239).

Table 3 reports the results of the Pearson correlation coefficients between the main variables. We can see that the correlation coefficient between *LnTotalNews* and *CR* is −0.3, indicating that bonds with higher credit ratings are exposed to more financial news. In addition, we find the negative/positive correlation between the bond yield spread and bad/good news, implying that bond investors may have negative/positive reactions to bad/good news.

## Results and Discussion

### Preliminary Findings

Firstly, we study whether CRAs and bond investors have different reactions to financial news on the internet. The empirical results are shown in Table 4.

**TABLE 4:** Result about how CRAs and bond investors react toward financial news on the internet.

	(1)	(2)	(3)	(4)	(5)	(6)	(7)	(8)
Variables	BR	BR	BR	BR	YieldSpread	YieldSpread	YieldSpread	YieldSpread
BadRatio	−0.013				26.315***			
	(−0.19)				(2.86)			
GoodRatio		0.003				−22.166***		
		(0.05)				(−3.02)		
DayBadRatio			0.015				26.249***	
			(0.23)				(3.01)	
DayGoodRatio				−0.012				−23.467***
				(−0.23)				(−3.31)
LnTotalNews	0.016	0.016	0.016	0.016	−2.655*	−2.781*	−2.886*	−2.642*
	(1.59)	(1.58)	(1.55)	(1.57)	(−1.68)	(−1.75)	(−1.83)	(−1.68)
CR					56.622***	56.433***	56.410***	56.380***
					(13.49)	(13.4)	(13.43)	(13.40)
Firm Control	Yes	Yes	Yes	Yes	Yes	Yes	Yes	Yes
Industry	Yes	Yes	Yes	Yes	Yes	Yes	Yes	Yes
Year	Yes	Yes	Yes	Yes	Yes	Yes	Yes	Yes
Observations	2,394	2,394	2,394	2,394	2,510	2,510	2,510	2,510
Adj *R*^2^	0.627	0.627	0.627	0.627	0.56	0.561	0.561	0.561

This table includes regressions to test how CRAs and bond investors react to financial news on the internet based on Stata 15. Standardized betas are reported and p-values are presented in parentheses. Symbols of ***, **, and * represent significance at the 1, 5, and 10% level, respectively.

Columns (1)–(4) of Table 4 report the results of CRA reactions. Results show that the coefficients of *BadRatio*, *GoodRatio*, *DayBadRatio*, and *DayGoodRatio* are insignificant, indicating that financial news on the internet does not influence CRAs. This is inconsistent with Tsai et al. (2010), who found that news impacted CRAs.

The results may be interpreted as follows. First, CRAs extract the credit rating fees from bond issuers, whereas bond issuers can choose any CRAs they prefer. In this case, it is not strange that CRAs have motivations to underestimate financial news on the internet to please bond issuers and maintain the market share (Xia, 2014). Second, the financial news from the internet contains high noise and potential biases, which could mislead the market participants (Chen and Haga, 2021). Therefore, CRAs keep calm and tend to not adjust credit ratings before they see official announcements.

Columns (5)–(8) of Table 4 report the results of bond investor reactions. Results indicate that bond investors have significant reactions to both bad and good news. On average, 1% increase in bad news leads to an increase in the bond yield spread by 0.26 BP (column 5), while 1% increase in good news drives the bond yield spread down by 0.22 BP (column 6), indicating that the bad news has a more significant influence on bond investors. In addition, the coefficient of *DayBadRatio* (26.249, with a *t*-value of 3.01) and *DayGoodRatio* (−23.467, with a *t*-value of −3.31) in columns (7)–(8) show a similar conclusion that bond investors have an asymmetric reaction to bad and good news.

Overall, the empirical results indicate that bond investors have a stronger reaction to bad news than good news, which is similar to Du (2020) who found that negative news had a stronger influence on Japan’s stock market. Our results are also consistent with studies in psychology that negative information has a stronger impact on impressions than positive information (Baumeister et al., 2001). A number of explanations account for the asymmetric reaction have been put forward. The negative news bias may result from evolution as attention to negative information makes survival easier, and increases the possibility of genetic inheritance (Baumeister et al., 2001). The negative news bias also could be explained by loss aversion that investors care more about a loss of utility than a gain of equal magnitude (Kahneman and Tversky, 2013).

### Further Analysis of Enterprise Ownership

It is not amazing that central SOEs enjoy a tremendous news advantage in China. For example, central SOEs are more likely to get media attention and be exposed to positive news. Therefore, we divide the news into two groups: central SOE news and other enterprise news. Then we perform the interaction analysis to study the heterogeneous reactions under different enterprise ownerships. Particularly, we add four interaction terms, *Dum_SOE × BadRatio*, *Dum_SOE × GoodRatio*, *Dum_SOE × DayBadRatio*, and *Dum_SOE × DayGoodRatio*, to capture the differential reactions. *Dum_SOE* is a dummy variable, which is equal to 1 when the news is about central SOEs, but 0 otherwise. Table 5 shows the empirical results.

**TABLE 5:** Interaction analysis.

	(1)	(2)	(3)	(4)
Variables	YieldSpread	YieldSpread	YieldSpread	YieldSpread
BadRatio	2.593			
	(0.18)			
Dum_SOE × BadRatio	30.919*			
	(1.75)			
GoodRatio		3.259		
		(0.29)		
Dum_SOE × GoodRatio		−32.737**		
		(−2.34)		
DayBadRatio			−5.498	
			(−0.41)	
Dum_SOE × DayBadRatio			40.429**	
			(2.42)	
DayGoodRatio				3.564
				(0.33)
Dum_SOE × DayGoodRatio				−34.796**
				(−2.56)
LnTotalNews	−2.580	−2.534	−2.625*	−2.414
	(−1.62)	(−1.59)	(−1.65)	(−1.52)
CR	56.358***	56.359***	56.282***	56.222***
	(13.40)	(13.41)	(13.42)	(13.38)
Firm Control	Yes	Yes	Yes	Yes
Bond Control	Yes	Yes	Yes	Yes
Rate Control	Yes	Yes	Yes	Yes
Industry	Yes	Yes	Yes	Yes
Year	Yes	Yes	Yes	Yes
Observations	2,510	2,510	2,510	2,510
Adj *R*^2^	0.561	0.561	0.561	0.562

This table includes regressions to test the interaction effects based on Stata 15. Dum_SOE is a dummy variable which is equal to 1 if the news is related to central SOEs, but 0 otherwise. Dum_SOE × BadRatio, Dum_SOE × GoodRatio, Dum_SOE × DayBadRatio, and Dum_SOE × DayGoodRatio capture the differential impact of enterprise ownership about the bad news and good news. Standardized betas are reported and p-values are presented in parentheses. Symbols of ***, **, and * represent significance at the 1, 5, and 10% level, respectively.

Column (1) shows that the coefficient of *Dum_SOE × BadRatio* is 30.919 with a *t*-value of 1.75, indicating that bond investors have stronger reactions to negative news about other enterprises.

Column (2) shows that the coefficient of *Dum_SOE × GoodRatio* is −32.737 with a significance at 10% level, indicating that bond investors strongly react to positive news about other enterprises. Columns (3)–(4) show a similar result that the coefficients of *Dum_SOE × DayBadRatio* and *Dum_SOE × DayGoodRatio* are 40.429 and −34.796, respectively, both significant at 5% level.

Next, we divide the sample into two subsamples: bonds issued by central SOEs and other enterprises. Then we conduct the regression analysis based on the two subsamples and compare the results with the interaction analysis. The empirical results are shown in Table 6.

**TABLE 6:** Investor reactions based on central SOEs and other enterprises.

	(1)	(2)	(3)	(4)	(5)	(6)	(7)	(8)
Variables	YieldSpread	YieldSpread	YieldSpread	YieldSpread	YieldSpread	YieldSpread	YieldSpread	YieldSpread
BadRatio	−2.972	26.209**						
	(−0.26)	(2.13)						
GoodRatio			5.378	−18.736**				
			(0.55)	(−1.97)				
DayBadRatio					−3.057	23.947**		
					(−0.28)	(2.09)		
DayGoodRatio							7.758	−20.111**
							(0.85)	(−2.17)
LnTotalNews	−2.150*	−0.192	−2.086*	−0.162	−2.133*	−0.309	−2.094*	−0.034
	(−1.78)	(−0.08)	(−1.71)	(−0.07)	(−1.75)	(−0.13)	(−1.74)	(−0.01)
CR	46.461***	74.632***	46.474***	74.623***	46.485***	74.594***	46.354***	74.552***
	(8.62)	(13.92)	(8.74)	(13.91)	(8.69)	(13.93)	(8.68)	(13.90)
Bond Control	Yes	Yes	Yes	Yes	Yes	Yes	Yes	Yes
Firm Control	Yes	Yes	Yes	Yes	Yes	Yes	Yes	Yes
Rate Control	Yes	Yes	Yes	Yes	Yes	Yes	Yes	Yes
Industry	Yes	Yes	Yes	Yes	Yes	Yes	Yes	Yes
Year	Yes	Yes	Yes	Yes	Yes	Yes	Yes	Yes
Observations	690	1,820	690	1,820	690	1,820	690	1,820
Adj *R*^2^	0.612	0.403	0.613	0.403	0.612	0.403	0.613	0.403

This table includes regressions to test how bond investors react to financial news on the internet based on Stata 15. The bonds in columns (1), (3), (5), and (7) are issued by central SOEs, whereas the bonds in columns (2), (4), (6), and (8) are issued by other enterprises. Standardized betas are reported and p-values are presented in parentheses. Symbols of ***, **, and * represent significance at the 1, 5, and 10% level, respectively.

In columns (1), (3), (5), and (7), the bonds are issued by central SOEs, and results show that the coefficients of *GoodRatio*, *BadRatio*, *DayGoodRatio*, and *DayBadRatio* are insignificant, indicating that bond investors are not influenced by the news about central SOEs.

In columns (2), (4), (6), and (8), the bonds are issued by other enterprises, and the results show that the coefficients of *GoodRatio*, *BadRatio*, *DayGoodRatio*, and *DayBadRatio* are all significant at 5% level, indicating that bond investors are more sensitive to the news about other enterprises than SOEs. This is also consistent with the results in Table 5.

Overall, our results confirm that there is heterogeneity in the psychological reaction where bond investors do not react to news about SOEs but to the news about other enterprises. Two reasons could explain our result. First, investors have adjusted their sensitivity to the news about central SOEs as too much news about central SOEs is released on the internet every day. Second, central SOEs could get strong support and implicit guarantees from governments (Huang et al., 2020a), implying that bonds issued by central SOEs are secure. Thus, market participants are not sensitive to the news about central SOEs.

### Further Analysis of Old News and New News

**FIGURE 1:**
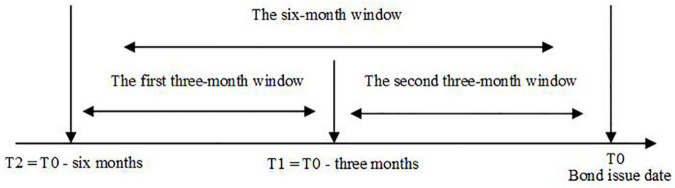
Dividing the 6-month window into two 3-month windows.

Some news can affect the bond investors for only a long period, whereas some news has a short-term impact on the bond investors. To examine whether both new news and old news have different influences on bond investors, we divide the 6-month window into two 3-month windows, which is shown in Figure 1.

In Figure 1, *T0* is the date when we issue the bond. We view the news in the first 3-month window as the past or old news, while news in the second 3-month window is viewed as the latest news or new news. Next, we construct the four new proxies to capture the news sentiment based on the two sub-windows, which is given as follow:


(6-1) $$D\,a\,y\,B\,a\,d\,R\,a\,t\,i\,o_{i}=\left\{ \begin{array}{c} \frac{T\,N\,D_{i}}{D_{i}}\,\,i\,f\,\,D_{i}>0\\ 0\,\,i\,f\,\,D_{i}=0\, \end{array}\right.$$

(6-2) $$D\,a\,y\,G\,o\,o\,d\,R\,a\,t\,i\,o_{i}=\left\{ \begin{array}{c} \frac{T\,P\,D_{i}}{D_{i}}\,\,i\,f\,\,D_{i}>0\\ 0\,\,i\,f\,\,D_{i}=0\, \end{array}\right.$$

(6-3) $$C\,D\,a\,y\,B\,a\,d\,R\,a\,t\,i\,o_{i}=\left\{ \begin{array}{c} \frac{T\,N\,D_{i}}{D}\,\,i\,f\,\,D>0\\ 0\,\,i\,f\,\,D=0\, \end{array}\right.$$

(6-4) $$C\,D\,a\,y\,G\,o\,o\,d\,R\,a\,t\,i\,o_{i}=\left\{ \begin{array}{c} \frac{T\,P\,D_{i}}{D}\,\,i\,f\,\,D>0\\ 0\,\,i\,f\,\,D=0\, \end{array}\right.$$

where *i* is equal to 1 if the news is released in the first 3-month window, but 2 in the second 3-month window. _*TND_i*_ is the total number of negative days in window *i.*_*TPD*_*i*__ is the total number of positive days in window *i*. _*D_i*_ is the total days in which the financial news is released on the internet for one specific enterprise in window *i*. *D* is the total days in which the financial news is released on the internet for one specific enterprise in the 6-month window.

Finally, we examine whether bond investors react to financial news based on the news in two sub-windows. The empirical results are shown in Table 7.

**TABLE 7:** Investor reaction to old news and new news.

	(1)	(2)	(3)	(4)	(5)	(6)
Variables	YieldSpread	YieldSpread	YieldSpread	YieldSpread	YieldSpread	YieldSpread
DayBadRatio_1_	15.978**					
	(2.16)					
DayGoodRatio_1_		−12.017**				
		(−2.19)				
LnTotalNews_1_	−2.930*	−1.919				
	(−1.86)	(−1.22)				
DayBadRatio_2_			15.738**			
			(2.14)			
DayGoodRatio_2_				−13.698**		
				(−2.53)		
LnTotalNews_2_			−2.640	−1.066		
			(−1.64)	(−0.65)		
CBadDayRatio_1_					23.641**	
					(2.11)	
CBadDayRatio_2_					29.521**	
					(2.23)	
CGoodDayRatio_1_						−22.341***
						(−2.74)
CGoodDayRatio_2_						−24.860***
						(−2.92)
LnTotalNews					−2.905*	−2.616*
					(−1.84)	(−1.65)
CR	56.513***	56.502***	56.396***	56.271***	56.392***	56.377***
	(13.44)	(13.41)	(13.41)	(13.40)	(13.42)	(13.40)
Firm Control	Yes	Yes	Yes	Yes	Yes	Yes
Bond Control	Yes	Yes	Yes	Yes	Yes	Yes
Rate Control	Yes	Yes	Yes	Yes	Yes	Yes
Industry	Yes	Yes	Yes	Yes	Yes	Yes
Year	Yes	Yes	Yes	Yes	Yes	Yes
Observations	2,510	2,510	2,510	2,510	2,510	2,510
Adj *R*^2^	0.560	0.560	0.560	0.560	0.561	0.561

This table includes regressions to test how bond investors react to old news and new news on the internet based on Stata 15. Standardized betas are reported and p-values are presented in parentheses. Symbols of ***, **, and * represent significance at the 1, 5, and 10% level, respectively.

Columns (1)–(2) show that the coefficients of *DayBadRatio*_1_ and *DayGoodRatio*_1_ are both significant at 5% level, indicating bond investors react to bad and good news in the first 3-month window. Columns (3)–(4) show a similar result that news in the second 3-month window also exhibits a significant influence on bond investors. In column (5), the coefficient of *CBadDayRatio*_2_ (29.251, with a *t*-value of 2.23) is large than the coefficient of *CBadDayRatio*_1_ (23.641, with a *t*-value of 2.11), indicating that the latest news rather than past news has a stronger influence on bond investors. A similar pattern is observed about the coefficients of *CGoodDayRatio*_2_ (−24.860, with a *t*-value of −2.92) and *CGoodDayRatio*_1_ (−22.341, with a *t*-value of −2.74) in column (6).

Overall, our results confirm the asymmetric response driven by news timeliness that bond investors are more sensitive to new news than old news, which is within our expectations. Two explanations account for this phenomenon. First, old news contains gossip or uncertain information, which may fluctuate investor emotions in the past (Chen and Haga, 2021). However, as time goes by, the information in old news may have no fundamental values, suggesting that the influence of old news on bond investors will decline over time. Second, new news contains more risk and uncertainty, leading an emotional decision (Stracca, 2004).

## Robust Check

### Alternative Measure of Bond Yield Spread

Previous section in our paper calculates the bond yield spread based on the nominal interest rate when the bond is issued. In the first robust check, we use the average closing price in the secondary bond market to estimate the bond yield spread. Then we again perform the regressions to test investor reactions to financial news on the internet. Table 8 presents the results.

**TABLE 8:** Robust check 1.

	(1)	(2)	(3)	(4)	(5)	(6)	(7)	(8)
Variables	YieldSpread	YieldSpread	YieldSpread	YieldSpread	YieldSpread	YieldSpread	YieldSpread	YieldSpread
BadRatio	36.394***				3.177			
	(2.74)				(0.23)			
GoodRatio		−27.767***				0.862		
		(−2.72)				(0.08)		
DayBadRatio			35.059***				1.720	
			(2.89)				(0.13)	
DayGoodRatio				−29.443***				3.738
				(−3.00)				(0.36)
LnTotalNews	−2.142	−2.006	−2.324	−1.832	−1.880	−1.803	−1.862	−1.766
	(−0.84)	(−0.79)	(−0.91)	(−0.72)	(−1.38)	(−1.31)	(−1.35)	(−1.31)
CR	61.533***	61.416***	61.436***	61.233***	45.326***	45.174***	45.248***	45.068***
	(11.00)	(10.97)	(11.01)	(10.94)	(7.77)	(7.86)	(7.82)	(7.82)
Firm Control	Yes	Yes	Yes	Yes	Yes	Yes	Yes	Yes
Bond Control	Yes	Yes	Yes	Yes	Yes	Yes	Yes	Yes
Rate Control	Yes	Yes	Yes	Yes	Yes	Yes	Yes	Yes
Industry	Yes	Yes	Yes	Yes	Yes	Yes	Yes	Yes
Year	Yes	Yes	Yes	Yes	Yes	Yes	Yes	Yes
Observations	1,629	1,629	1,629	1,629	653	653	653	653
Adj *R*^2^	0.496	0.496	0.496	0.496	0.571	0.571	0.571	0.571

This table includes regressions to test how CRAs and bond investors react to financial news on the internet based on Stata 15. Standardized betas are reported and p-values are presented in parentheses. Symbols of ***, **, and * represent significance at the 1, 5, and 10% level, respectively.

In columns (1)–(4), the bonds are issued by other enterprises. We observe that the coefficients of *BadRatio*, *GoodRatio*, *DayBadRatio*, and *DayGoodRatio* are significant at 1% level. In addition, the absolute value of the coefficients of *BadRatio* and *DayBadRatio* are relatively larger than the coefficients of *GoodRatio* and *DayGoodRatio*. Conversely, the bonds in columns (5)–(6) are issued by central SOEs, and we find that the coefficients of news sentiment variables are insignificant. Overall, the result confirms our previous conclusions.

### Alternative Measure of News Sentiment

The method in our empirical study to classify news may misclassify news type. In this subsection, we follow Chen et al. (2021) and measure the news sentiment as follow:


(7) $$d\,i\,v=\log\frac{1+N}{1+P}$$

where *N* and *P* are the total number of negative and positive words for a specific enterprise.

Then we again perform the regression based new news sentiment variable. Table 9 presents the results. In columns (1)–(5), we calculate *div* based on the 6-month window. In column (6), *div1* and *div2* are calculated based on the first and second 3-month window, respectively.

**TABLE 9:** Robust check 2.

	(1)	(2)	(3)	(4)	(5)	(6)
Variables	BR	YieldSpread	YieldSpread	YieldSpread	YieldSpread	YieldSpread
div	0.007	13.040***	−1.693	17.258***		
	(0.33)	(4.83)	(−0.46)	(5.49)		
div1					6.909***	
					(2.99)	
div2						7.666***
						(3.32)
LnTotalNews	−0.001	−2.388	−2.221*	−1.106	−1.382	−1.736
	(−0.08)	(−1.52)	(−1.91)	(−0.48)	(−0.85)	(−1.10)
CR		56.124***	46.439***	61.105***	56.184***	56.235***
		(13.33)	(8.75)	(11.66)	(13.37)	(13.35)
Firm Control	Yes	Yes	Yes	Yes	Yes	Yes
Bond Control	No	Yes	Yes	Yes	Yes	Yes
Rate Control	No	Yes	Yes	Yes	Yes	Yes
Industry	Yes	Yes	Yes	Yes	Yes	Yes
Year	Yes	Yes	Yes	Yes	Yes	Yes
Observations	2,394	2,510	690	1,820	2,510	2,510
Adj *R*^2^	0.624	0.563	0.612	0.491	0.561	0.561

This table includes regressions to test how CRAs and bond investors react to financial news on the internet based on Stata 15. Standardized betas are reported and p-values are presented in parentheses. Symbols of ***, **, and * represent significance at the 1, 5, and 10% level, respectively.

Column (1) shows that the coefficient of *div* is insignificant, indicating that CRAs have no reactions to financial news on the internet. In columns (2)–(4), bonds are issued by all listed enterprises, central SOEs, and other enterprises, respectively. It is observed that the coefficients of *div* are negative in column (2) and column (4), both significant at 1% level, while the coefficient of *div* in column (3) is insignificant, indicating that bond investors are sensitive to the news about other enterprises but not to the news about central SOEs. Columns (5)–(6) show that the coefficients of *div1* and *div2* are 6.909 and 7.666, both significant at 1% level, suggesting that both old and new news influence bond investors, but bond investors have strong reactions to new news.

### Endogenous Test

We also employ the instrumental variables (IVs) and conduct a two-stage least square (2SLS) estimation to avoid the endogenous problem. We introduce two variables that are theoretically non-related to the bond yield spread and bond rating but associated with *DayBadRatio*, *DayGoodRatio*, *BadRatio*, and *GoodRatio* as our IVs. These IVs are *IV_BadRatio*, *IV_GoodRatioIV*, *IV_BadRatio*, and *IV_GoodRatio*, which are the average of *DayBadRatio*, *DayGoodRatio*, *BadRatio*, and *GoodRatio* of other bond issuers with the same year, industry, corporate entity rating. A larger IV captures a higher likelihood of exposure to the medium, reflecting that bond issuers with similar performance and industry could get similar media attention. Next, we employ a first-stage probit model, as specified in Model (8), to estimate the predicted values of *DayBadRatio*, *DayGoodRatio*, *BadRatio*, and *GoodRatio*, respectively, which are then used as an instrument in the second-stage least squares estimation.


$$X=\alpha +\beta_{0}\,I\,V\,X+\beta_{1}\,L\,n\,T\,o\,a\,l\,N\,e\,w\,s+F\,i\,r\,m\,C\,o\,n\,t\,r\,o\,l+$$

(8) I⁢n⁢d⁢u⁢s⁢t⁢r⁢y+Y⁢e⁢a⁢r+ε

where *X* is *DayBadRatio*, *DayGoodRatio*, *BadRatio*, or *GoodRatio*, *IVX* is our IV candidates, which are *IV_BadRatio*, *IV_GoodRatioIV*, *IV_BadRatio*, or *IV_GoodRatio*.

Table 10 presents the 2SLS regression results. Columns (1), (3), (5), and (7) report the first-stage regression results, and we find the significant correlation between the four news indexes (*DayBadRatio*, *DayGoodRatio*, *BadRatio*, and *GoodRatio*) and our IV candidates (*IV_BadRatio*, *IV_GoodRatioIV*, *IV_BadRatio*, and *IV_GoodRatio*), thereby proving our instrumental variables’ relevancy. Columns (2), (4), (6), and (8) present the second-stage regression results, where *DayBadRatio, DayGoodRatio, BadRatio, and GoodRatio* in the baseline regression are replaced with the predicted value, *Hat_DayBadRatio, Hat_DayGoodRatio, Hat_BadRatio, and Hat_GoodRatio*, from the first-stage regression. Column (2) and column (6) show that the coefficient of *Hat_DayBadRatio* (coefficient: 68.436; *t*-value: 3.51) and *Hat_BadRatio* (coefficient: 67.202; *t*-value: 3.26) remains positive and significant at 1% level. Column (4) and column (8) show a similar results that the coefficients of *Hat_DayGoodRatio* (coefficient: −43.243; *t*-value: 2.75) and *Hat_GoodRatio* (coefficient: −38.93; *t*-value: −2.38) are significant. Overall, these findings confirm that bad (good) news can significantly affect the bond investor reactions, consistent with our previous results.

**TABLE 10:** Robust check 3.

	(1)	(2)	(3)	(4)	(5)	(6)	(7)	(8)

	**Stage 1**	**Stage 2**	**Stage 1**	**Stage 2**	**Stage 1**	**Stage 2**	**Stage 1**	**Stage 2**
**Variables**	**DayBadRatio**	**YieldSpread**	**DayGoodRatio**	**YieldSpread**	**BadRatio**	**YieldSpread**	**GoodRatio**	**YieldSpread**
Hat_DayBadRatio		68.436***						
		(3.51)						
Hat_DayGoodRatio				−43.243***				
				(−2.75)				
Hat_BadRatio						67.202***		
						(3.26)		
Hat_GoodRatio								−38.930**
								(−2.38)
IV_DayBadRatio	0.960***							
	(25.90)							
IV_DayGoodRatio			0.975***					
			(31.78)					
IV_BadRatio					0.967***			
					(24.58)			
IN_GoodRatio							0.971***	
							(29.60)	
LnTotalNews	0.014***	−3.660**	−0.007	−2.844*	0.011***	−3.282**	−0.013***	−3.084*
	(3.94)	(−2.20)	(−1.49)	(−1.74)	(3.13)	(−1.99)	(−2.79)	(−1.87)
CR	0.005	55.721***	−0.016**	55.904***	0.002	55.920***	−0.015**	56.004***
	(0.85)	(18.78)	(−2.25)	(18.82)	(0.29)	(18.86)	(−2.16)	(18.84)
Firm Control	Yes	Yes	Yes	Yes	Yes	Yes	Yes	Yes
Bond Control	No	Yes	No	Yes	No	Yes	No	Yes
Rate Control	No	Yes	No	Yes	No	Yes	No	Yes
Industry	Yes	Yes	Yes	Yes	Yes	Yes	Yes	Yes
Year	Yes	Yes	Yes	Yes	Yes	Yes	Yes	Yes
Observations	2,554	2,510	2,554	2,510	2,554	2,510	2,554	2,510
Adj *R*^2^	0.259	0.561	0.252	0.560	0.262	0.561	0.256	0.560

This table includes 2SLS regressions to test bond investors’ reactions to financial news on the internet based on Stata 15. Column (1), column (3), column (5), and column (7) show the first-stage results, whereas column (2), column (4), column (6) and column (8) show the second-stage results. IV_DayBadRatio, IV_DayGoodRatio, IV_BadRatio, and IN_GoodRatio are four IVs. Hat_DayBadRatio, Hat_DayGoodRatio, Hat_BadRatio, and Hat_GoodRatio are four predicted values from the first-stage regressions. Standardized betas are reported and p-values are presented in parentheses. Symbols of ***, **, and * represent significance at the 1, 5, and 10% level, respectively.

## Conclusion

This article attempts to test the psychological reactions of CRAs and bond investors to the financial news on the internet in China’s bond market. As we know, much negative and credible news could be observed on the internet before the bond defaults, but CRAs do not downgrade the bond issuer. On the other hand, the bond price may fall when some negative news about the bond issuer is released on the internet. These phenomena indicate that CRAs and bond investors may have different psychological reactions to financial news. Therefore, we attempt to examine how CRAs and bond investors react to financial news on the internet.

Our study has several interesting findings. First, it demonstrates that CRAs and bond investors react differently to financial news on the internet. Specifically, CRAs display a tendency to underreact to financial news, consistent with the phenomenon that bonds exposed to bad financial news could still receive high credit ratings. However, there exists an asymmetry in psychological reactions where bond investors react stronger to bad news than to good news, consistent with the studies in behavioral finance and psychology. Second, bond investors’ reactions to financial news with heterogeneity that is reflected in the enterprise ownership and news timeliness. Specifically, investors react to news about central enterprises but not news about other enterprises. In addition, investors are more sensitive to new news than old news. Overall, the study’s practical contribution helps regulators obtain bond participants’ reactions to financial news and formulate corresponding regulatory rules quickly. Our research also provides a reference for CRAs and investors to know psychological reactions so that they avoid overreacting or underreacting to the news.

This study has several limitations. One limitation is that we conduct our empirical study based on the listed enterprises in China, which limits its generalizability. Bonds issued by unlisted enterprises are important parts of the bond market. Usually, unlisted enterprises receive little media attention and relatively low credit ratings, and Defond and Zhang (2014) argued that bond investors were more sensitive to junk or low-rated bonds. Therefore, bond investors may have heterogeneous reactions to news about listed and unlisted enterprises. Another limitation is that we do not classify the types of news in detail. For example, Shepperd and McNulty (2002) revealed that investors reacted differently to the expected and unexpected news. However, we do not distinguish between expected and unexpected news in this study. In the future, we will study how CRAs and bond investors react to expected and unexpected financial news on the internet based on the listed and unlisted enterprises.

## Data Availability Statement

The raw data supporting the conclusions of this article will be made available by the authors, without undue reservation.

## Author Contributions

WZ designed, drafted, and revised the manuscript. JW collected the data, designed the study, and revised the manuscript. MT designed, reviewed, and revised the manuscript. All authors contributed to the article and approved the submitted version.

## Conflict of Interest

The authors declare that the research was conducted in the absence of any commercial or financial relationships that could be construed as a potential conflict of interest.

## Publisher’s Note

All claims expressed in this article are solely those of the authors and do not necessarily represent those of their affiliated organizations, or those of the publisher, the editors and the reviewers. Any product that may be evaluated in this article, or claim that may be made by its manufacturer, is not guaranteed or endorsed by the publisher.

## Footnotes

1 If there is an indistinguishable situation in the voting process, such as one positive vote, one neutral vote, and one negative vote, we will manually determine the final result.

2
https://www.wind.com.cn/
